# Heparin at physiological concentration can enhance PEG-free *in vitro* infection with human hepatitis B virus

**DOI:** 10.1038/s41598-017-14573-9

**Published:** 2017-10-31

**Authors:** Gansukh Choijilsuren, Ren-Shiang Jhou, Shu-Fan Chou, Ching-Jen Chang, Hwai-I Yang, Yang-Yuan Chen, Wan-Long Chuang, Ming-Lung Yu, Chiaho Shih

**Affiliations:** 1 Taiwan International Graduate Program in Molecular Medicine, National Yang-Ming University and Academia Sinica, Taipei, Taiwan; 2 Institute of Biomedical Sciences, Academia Sinica, Taipei, Taiwan; 3 Institute of Biochemistry and Molecular Biology, National Yang-Ming University, Taipei, Taiwan; 4 Genomics Research Center, Academia Sinica, Taipei, Taiwan; 5 Changhua Christian Hospital, Changhua, Taiwan; 6 Hepatobiliary Division, Department of Internal Medicine, Kaohsiung Medical University Hospital, Kaohsiung, Taiwan

## Abstract

Hepatitis B virus (HBV) is a blood-borne pathogen responsible for chronic hepatitis, cirrhosis, and liver cancer. The mechanism of HBV entry into hepatocytes remains to be investigated. Recently, sodium taurocholate cotransporting polypeptide (NTCP) was discovered as a major HBV receptor based on an *in vitro* infection system using NTCP-reconstituted HepG2 cells. However, this infection system relies on the compound polyethylene glycol (4% PEG), which is not physiologically relevant to human infection. High concentration of heparin has been commonly used as an inhibitor control for *in vitro* infection in the field. Surprisingly, we found that heparin at physiological concentration can enhance HBV infection in a PreS1-peptide sensitive, NTCP-dependent manner in both HepaRG and HepG2-NTCP-AS cells. O-sulfation of heparin is more important for the infection enhancement than N-sulfation. This system based on the HepG2-NTCP-AS cells can support *in vitro* infection with HBV genotypes B and C, as well as using serum samples from HBeAg positive and negative chronic carriers. In summary, our study provides a PEG-free infection system closely resembling human natural infection. In addition, it points to a future research direction for heparin and heparin-binding host factor(s) in the blood, which are potentially involved in viral entry. To our knowledge, this is the first soluble and circulatory host factor which can enhance HBV in vitro infection.

## Introduction

HBV is an enveloped and partially double-stranded DNA virus which established chronic infection in around 240 million carriers worldwide. Current treatment for HBV cannot effectively eradicate the virus from chronic hepatitis B patients^1^. These patients have a high risk to develop liver cirrhosis and hepatocellular carcinoma^2^. Studies on the HBV life cycle have been hampered by the lack of an efficient and user-friendly *in vitro* infection system. Primary human hepatocytes (PHHs) and HepaRG cells had served as valuable tools for studying the early event of viral entry, albeit the viral receptor remained elusive^3–5^. One major concern of primary human hepatocytes is its expensive cost from the commercial source. In addition, it is not a robust system because the qualities of hepatocytes tend to vary from lot to lot. In contrast, the HepaRG cell system is relatively more reliable and consistent. However, the infection efficiency is not high, and it involves tedious work to grow and maintain well-differentiated HepaRG cells^5^. Recently, sodium taurocholate cotransporting polypeptide (NTCP) was identified as a functional receptor for HBV and HDV^6^. This NTCP-reconstituted HepG2 system has emerged as a powerful resource in the field of HBV research^7–9^.

One common way to achieve a higher efficiency in HBV infection is to include 4–5% of polyethylene glycol (PEG) during the incubation period of viral infection. For example, in the presence of PEG, the efficiency of HBV infection on PHH was elevated up to 20 times, mainly due to the enhanced adsorption between virus and hepatocytes^4^. Similarly, PEG was able to promote HBV infection in HepaRG and NTCP-reconstituted HepG2 hepatocytes^5,6^. On the other hand, the use of PEG is not without any reservations. For example, PEG is known to induce membrane fusion, such as its use in the generation of hybridoma^10,11^. Therefore, PEG might provoke non-specific fusion between the virions and the host cell membrane. Most importantly, PEG is a non-biological chemical not found in human body. It remains a concern whether the current PEG-containing infection protocol could faithfully mimic the viral entry event *in vivo*.

HBV infection is supposed to be initiated by HBV binding to the heparan sulfate proteoglycans (HSPGs) on the hepatocyte surface^12^. Soluble heparins, which share a similar structure with HSPGs, could therefore inhibit HBV entry by interrupting the interaction between HBV and HSPGs on target cells^12^. As a highly sulfated glucosaminoglycan, heparin is mainly secreted by basophils and mast cells^13^. The normal physiological concentration of heparin in human plasma ranges from 1 to 5 μg/ml^14–16^. The physiological roles of heparin and HSPGs are highly diverse, including anticoagulation, signaling, development, anti-inflammation and anti-metastatic^17,18^. To date, more than 400 heparin-binding proteins have been reported in literature^19^.

In this study, we revisited the effects of heparin and PEG on HBV infection. Consistent with a general impression about heparin in HBV infection, we also found that heparin at a higher concentration, can inhibit HBV infection^12,20–22^. However, to our surprise, at its physiological concentration (1 to 5 μg/ml), heparin could actually enhance HBV infection efficiency in both HepaRG and NTCP-reconstituted hepatocytes. This phenomenon was most pronounced when without PEG or with a reduced amount of PEG (1.2%). This enhancement effect of heparin could become obscured in the presence of 4% PEG, a concentration commonly used in HBV *in vitro* infection experiments. Our study established a PEG-free *in vitro* infection system which resembles more closely the physiological condition in the liver. One implication from our studies here is that heparin-binding host factors may deserve further attention in future studies of viral entry.

## Results

### Establishment of an HBV *in vitro* infection system using a HepG2-NTCP-AS cell line

**Figure 1:**
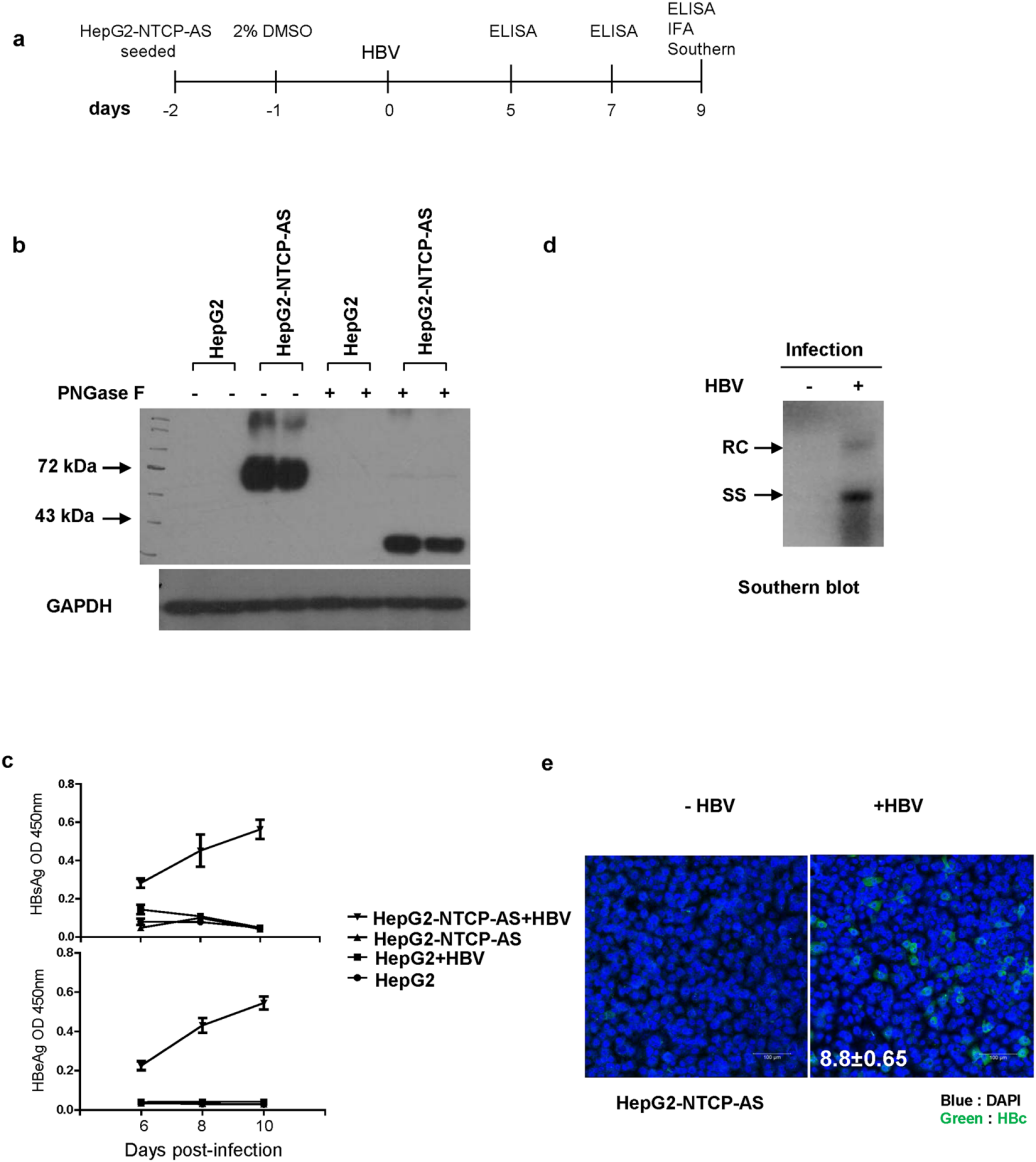
Establishment of an HBV *in vitro* infection system using a HepG2-NTCP-AS cell line. (**a**) An illustration of experimental design. (**b**) The expression of Flag-tagged NTCP in a HepG2-NTCP-AS stable cell line was detected by Western Blot using an anti-Flag antibody. De-glycosylation of NTCP by the PNGase F treatment shifted the MW from around 72 kD to 36  kD. The parental HepG2 cells served as a negative control for Flag-NTCP expression. GAPDH expression served as a loading control. (**c**) Similar trends of increasing expressions of both HBsAg and HBeAg were detected by ELISA only in HepG2-NTCP-AS cells infected with human HBV-containing serum. No detectable increase in HBsAg and HBeAg was noted in the negative controls using HepG2 cells or mock infection with no virus. (**d**) Total intracellular core particle-associated viral DNAs were analyzed by Southern blot at 9 dpi. Non-infected HepG2-NTCP-AS cells served as a negative control. RC: relaxed circle DNA; SS: single-strand DNA. (**e**) Confocal microscopy detected HBc protein (green) in HepG2-NTCP-AS cells infected with serum-derived HBV. Cell nuclei were counterstained with DAPI (blue).

The experimental procedure for an HBV *in vitro* infection is as outlined in Fig. 1a. To establish an NTCP-expressing HepG2 cell line for *in vitro* infection with HBV, HepG2 cells were stably transfected with a human NTCP-flag expression plasmid driven by a CMV promoter. A stable clone with the highest level of NTCP protein expression was named HepG2-NTCP-AS in this study. Glycosylated NTCP-flag protein is around 72 kD as detected by Western blot (Fig. 1b). Treatment with PNGase F removed the glycan from NTCP resulting in a 36 kD protein. We tested the infection efficiency of this HepG2-NTCP-AS cell line by using serum samples from HBV carriers (Supplementary Table S1) in the presence of 4% PEG. The serum sample B76 containing the highest HBV DNA titer was chosen for use in this study. In the time course experiments, increasing amounts of HBsAg (HBV surface antigen) and HBeAg (HBV e antigen) can be detected in the media by ELISA at various time points post-infection (Fig. 1c). No signals of HBsAg and HBeAg were detected in the negative control of parental HepG2 cells containing no NTCP. The trends of increasing levels of HBsAg and HBeAg in the post-infection time course strongly supports for the *de novo* synthesis of HBsAg and HBeAg in the infected HepG2-NTCP-AS cells. Consistent with the result from the ELISA assay, HBV replicative DNA intermediates can be detected on day 9 post-infection by Southern blot analysis (Fig. 1d). Moreover, expression of HBV core protein (HBc) can be visualized in 8–9% of infected cells by immunostaining and confocal microscopy using anti-HBc antibody (Fig. 1e). No HBc was detected in mock-infected cells. Taken together, our data provided evidence that we have successfully established an HBV *in vitro* infection system based on the NTCP reconstituted HepG2 cells. Furthermore, this HepG2-NTCP-AS cell system can support *in vitro* infection using serum samples from both HBeAg positive and negative patients, as well as using input viruses from HBV genotypes B and C (Supplementary Table S1; Fig. S1a). HBV DNA from the HBeAg-negative patient contains a hotspot stop codon mutation at precore codon 28, by PCR cloning and sequencing (Fig. S1b).

### Heparin at physiological concentration in human plasma can stimulate HBV *in vitro* infection

While NTCP has been identified as a receptor for HBV entry^6^, it is possible that other unknown host factors could also contribute to HBV viral entry. For example, hNTCP transgenic mice cannot support *in vivo* HBV infection or re-infection^9^. To look for any soluble host factors which might facilitate HBV infection of hepatocytes, we examined the plasma from a healthy individual for its potential effect on HBV *in vitro* infection. HepG2-NTCP-AS cells were infected with serum from an HBV chronic carrier (B76), in the presence or absence of 10% human plasma. However, dilution of human sodium citrated plasma in DMEM medium resulted in plasma clotting due to the diluted sodium citrate (anticoagulant) in human plasma. This clotting issue cannot be circumvented by replenishing sodium citrate to the infectious inoculum due to its calcium-chelating properties against cell attachment on the dish. To prevent plasma from clotting in cell culture, we mixed heparin into the infectious inoculum. Heparin has been known for its potent anticoagulant activity^23^. Unfortunately, it was also widely used as an HBV entry inhibitor^12,20–22^. To minimize the inhibitory effect of heparin on HBV *in vitro* infection, we used 4.5 μg/ml heparin as a minimum anticoagulant concentration in the infectious inoculum. Another unexpected problem in plasma clotting is about the use of 4% PEG. In the field, 4% PEG has been routinely included to enhance HBV *in vitro* infection efficiency^4,12,20–22,24^. Unfortunately, 4% PEG also promoted plasma clotting. Therefore, we reduced the PEG concentration down to 1.2% in the infectious inoculum in our experimental procedure of *in vitro* infection.

**Figure 2:**
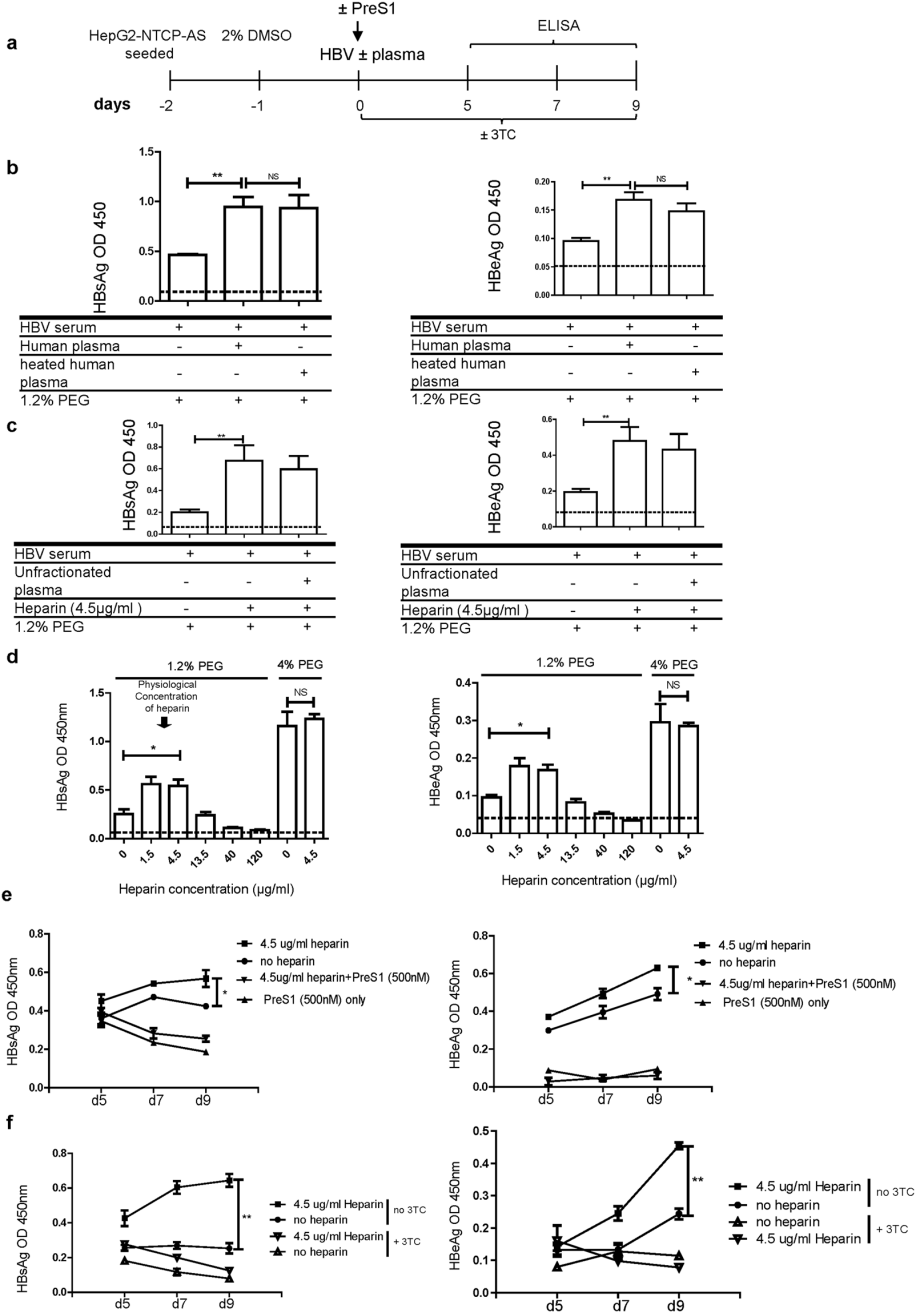
Heparin at physiological concentration in human plasma can stimulate HBV *in vitro* infection. (**a**) An illustration of experimental design. (**b**) Human plasma, with or without heat pre-treatment, enhanced HBV *in vitro* infection. HBsAg (left panel) and HBeAg (right panel) were measured at 9 dpi by ELISA. HepG2-NTCP-AS cells were infected with HBV serum at MOI 300–3000 in the presence of 1.2% PEG. Human plasma was supplemented with fresh heparin before adding to the cell culture to prevent coagulation. Data are shown as mean ± SEM of at least three independent experiments (**p < 0.01). Dashed lines represent the cutoff value of the background noise in the ELISA assays. (**c**) Heparin alone without human plasma can still enhance HBV infection. (**d**) Heparin at physiological concentrations (1 to 5 µg/ml)^14–16^ can enhance HBV infection in a dose-response experiment. HepG2-NTCP-AS cells were infected with HBV serum at MOI 300–3000 at 37 °C for 24 hours. Increasing concentrations of heparin (0, 1.5, 4.5, 13.5, 40, 120 μg/ml) were used in the presence of 1.2% PEG or 4% PEG. (*p < 0.05). (**e**) Treatment with PreS1 lipopolypeptide inhibited heparin-enhanced infection of HepG2-NTCP-AS cells. These HepG2-NTCP-AS cells were infected with HBV serum in the presence of 1.2% PEG with and without 4.5 μg/ml heparin. HepG2-NTCP-AS cells were pre-incubated with 500 nM PreS1 peptide for 30 minutes before infection. (*p < 0.05). (**f**) Heparin-enhanced HBV infection can be abrogated by the continuous presence of a nucleoside analog 3TC (10 uM), from 1 to 9 dpi. HepG2-NTCP-AS cells were infected with HBV serum in the presence of 1.2% PEG, with and without 4.5 μg/ml heparin.

As outlined in Fig. 2a, we performed *in vitro* infection assay in search for potential enhancement factor(s) in human plasma. Interestingly, we detected reproducibly increased levels of HBsAg and HBeAg, when the infection experiment was supplemented with human plasma, compared to the serum only control (no plasma) (Fig. 2b). This phenomenon was observed whether or not human plasma was pretreated with heat. We fractionated human plasma from a healthy individual by Amicon Ultra centrifugal filters according to the molecular weights of 100 kD, 10 kD, and 3 kD. Plasma fractions do not clot due to the disrupted coagulation cascade. Different MW fractions of plasma, with or without heparin supplementation, were tested for HBV infection. To our surprise, none of these plasma fractions had any significant effect on HBV infection, if without heparin supplementation (Fig. S2a). In contrast, when heparin was supplemented during HBV infection, all fractions of human plasma exhibited an enhancing effect on HBV infection (Fig. S2b). These results led us to the hypothesis that heparin itself at lower concentrations might have an enhancing effect on HBV infection. We compared further the heparin effect without human plasma, and observed the infection enhancement in a plasma-independent manner (Fig. 2c). Our finding contradicts the current knowledge of heparin as a potent inhibitor of HBV infection^12,20–22^.

To resolve this apparent discrepancy in the heparin effect on HBV infection, we titrated for the optimum concentration of heparin in the range from 0 to 120 µg/ml (Fig. 2d). We observed good enhancement for infection when heparin concentration was around 1.5–4.5 μg/ml, and at 40 μg/ml, heparin showed 50% inhibition of HBV infection in this HepG2-NTCP-AS cell system. Similar dose-response profiles were detected between HBsAg and HBeAg assays (Fig. 2d). The enhancement effect of heparin on HBV infection was most pronounced when the amount of PEG was reduced. At 4% PEG, heparin effect was then masked by the stronger effect from 4% PEG, suggesting that the mechanism of heparin enhancement could also be at the initial step of adsorption and attachment to the heparan sulfate proteoglycan (HSPG) on the cell surface of hepatocytes^4,25^. This heparin-mediated enhancement on HBV infection is most likely to be NTCP-dependent, since the PreS1 treatment (Myrcludex B) resulted in complete inhibition of HBV infection (Fig. 2e). Next, we examined whether 3TC (lamivudine, a nucleoside analog) treatment could inhibit heparin enhancement on HBV infection. Continuous presence of 3TC was included in the medium between HBV inoculation and day 9 post-infection (Fig. 2a). Significant differences in both HBsAg and HBeAg secretion were detected between treatments with vs. without 3TC (Fig. 2f). The abolishment of the heparin enhancement on HBV infection by 3TC treatment, provided experimental evidence for *de novo* synthesis of HBsAg and HBeAg in this HepG2-NTCP-AS *in vitro* infection system.

Altogether, our studies on human plasma uncovered unexpectedly an enhancement effect of heparin, a naturally occurring anticoagulant in the blood, at its physiological concentration.

### Heparin facilitates PEG-free *in vitro* infection with HBV

**Figure 3:**
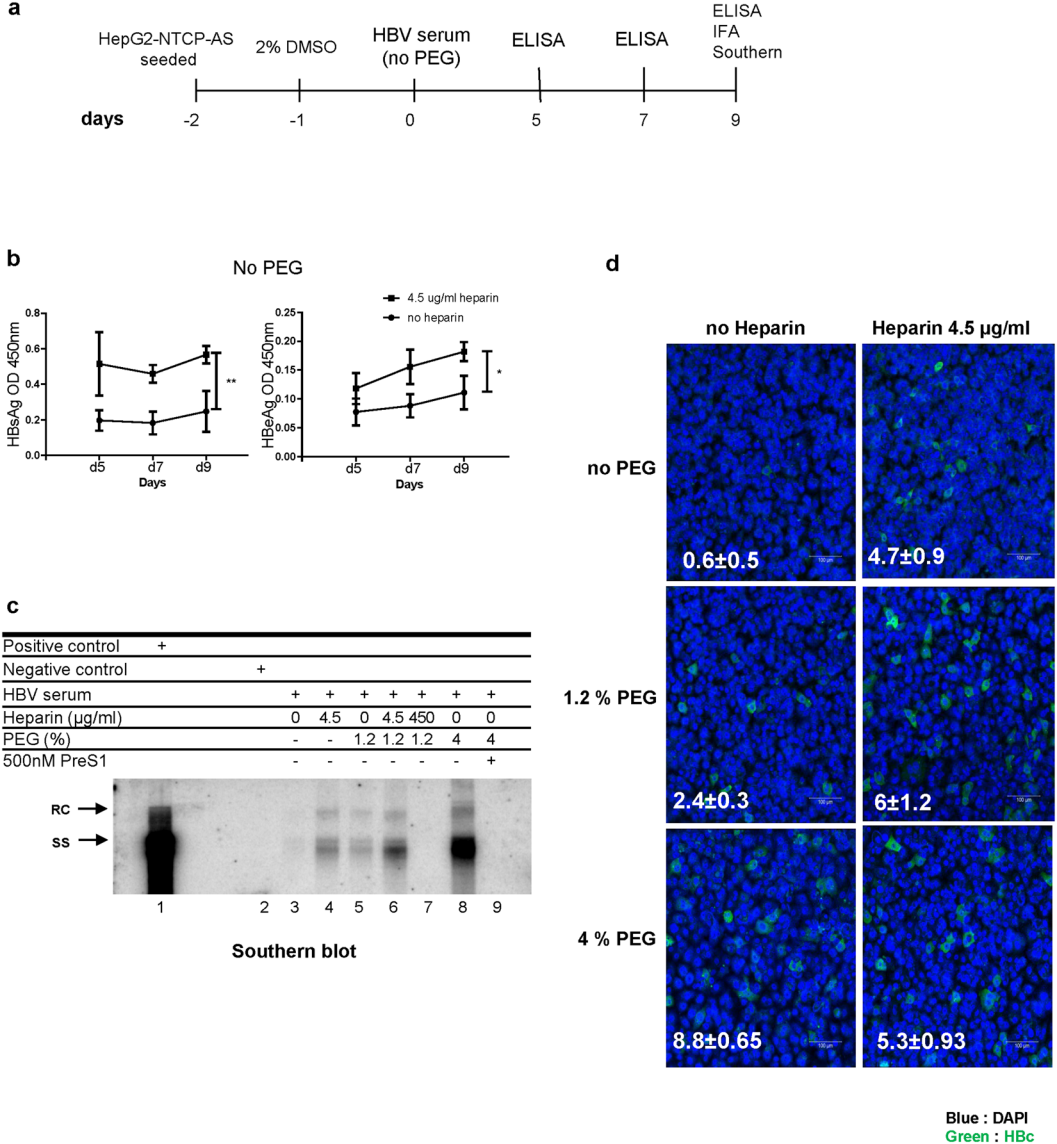
Heparin facilitates PEG-free *in vitro* infection with HBV. (**a**) An illustration of experimental design. (**b**) Heparin alone can enhance HBV infection in a PEG-independent manner. The secretions of HBsAg and HBeAg were measured every other day by ELISA in a time course experiment (1–9 dpi). HepG2-NTCP-AS cells were infected with HBV serum pre-mixed with and without 4.5 μg/ml heparin. (**c**) A combinatory effect of heparin (4.5 µg/ml) and PEG (1.2%) can be detected by Southern blot analysis. Lane 1 and 2: Positive and negative controls were prepared from HBV DNA transfected HuH-7 cells and mock-infected HepG2-NTCP-AS cells, respectively. Heparin at a lower dose (4.5 μg/ml) enhanced HBV *in vitro* infection in the presence of 1.2% PEG (compare lanes 5 and 6), or in the absence of PEG (compare lanes 3 and 4). However, overdosed heparin (450 μg/ml) or treatment with PreS1 peptide (500 nM) was strongly inhibitory to HBV infection (lane 7 and 9). 4% PEG exhibited the most potent effect on HBV infection (lane 8). (**d**) In a PEG-free *in vitro* infection system, low dose heparin (4.5 µg/ml) significantly increased the percentage of HBc-positive HepG2-NTCP-AS cells by confocal microscopy. Cell nucleus was counterstained with DAPI. The estimated percentages of HBc positivity were based on the average from three different microscopic fields (Mean ± SD). Approximately 500 cells were scored in each field.

We observed the heparin enhancement in the presence of 1.2% PEG, but not at 4% PEG (Fig. 2). Here, we asked whether heparin can enhance HBV infection without PEG (Fig. 3a). Indeed, even in the absence of PEG, an increase of HBV viral antigen expression was observed, when infectious inoculum was supplemented with 4.5 μg/ml heparin (Fig. 3b). The results of Southern blot analysis were consistent with those from the ELISA analysis. HBV infection with neither heparin nor PEG showed no detectable HBV-specific replication signal (lane 3, Fig. 3c). However, heparin treatment strikingly increased HBV infection efficiency as measured by Southern blot analysis (compare lane 3 and lane 4, Fig. 3c). Infection efficiency was further increased in combination treatment with 1.2% PEG and 4.5 μg/ml heparin, relative to 1.2% PEG treatment alone (compare lane 5 and lane 6, Fig. 3c). Heparin treatment at high concentration (450 µg/ml) completely abolished the signal of HBV replicative intermediates (compare lane 6 and lane 7, Fig. 3c). This result is consistent with the known inhibitory effect of heparin on HBV infection in literature^12,20–22^. The results of heparin enhancement by ELISA and Southern blot analyses (Fig. 3b and c) were confirmed by immunofluorescence assay of HBV core protein (Fig. 3d). Combination of 4.5 μg/ml heparin and 1.2% PEG increased the percentage of HBc positive cells (Fig. 3d, left vs. right panels), compared to those with no heparin treatment in the absence or the presence of 1.2% PEG. We noted that heparin reduced the percentage of HBc positive cell number in the presence of 4% PEG (Fig. 3d, bottom).

Heparin and heparan sulfate contain modifications like N-sulfation, N-acetylation or O-sulfation^26^. To determine whether heparin sulfation is required for heparin-enhancement on HBV infection, we compared the effects among sulfated heparin, O-desulfated (deO) heparin, and N-desulfated (deN) heparin using this HepG2-NTCP-AS-based *in vitro* infection assay (Fig. S3). While the N-desulfated heparin still maintained significant effect on HBV infection, O-desulfated heparin could no longer enhance HBV infection by the ELISA assay for HBsAg and HBeAg.

**Figure 4:**
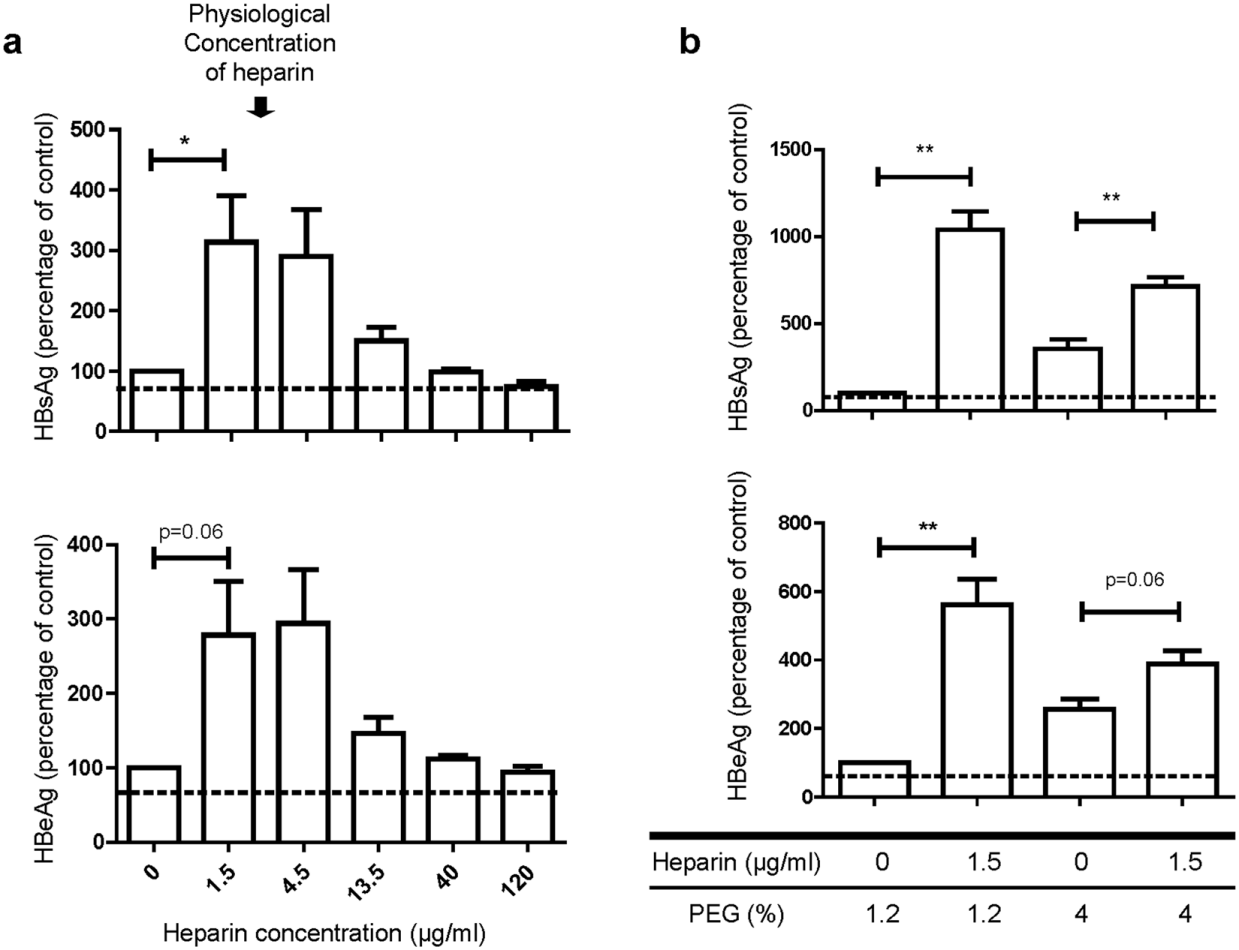
Heparin enhancement on HBV infection using the HepG2-NTCP-AS cells were validated by using a HepaRG infection system. (**a**) A dose-dependent curve of heparin on HBV *in vitro* infection using human HBV-containing serum and adherent HepaRG cells. Increasing concentrations of heparin treatment (0, 1.5, 4.5, 13.5, 40, 120 μg/ml) were tested in the presence of 1.2% PEG. (**b**) Heparin can further enhance HBV infection using HepaRG cells in the presence of 1.2% or 4% PEG.

Encouraged by the results of heparin-mediated enhancement on HBV infection, we asked if the same heparin enhancement can be observed in hepatocytes other than the HepG2-NTCP-AS cells. As shown in Fig. 4, the same stimulatory effect of heparin on HBV infection can be observed in another human hepatoma cell line HepaRG^5^. Moreover, O-sulfation of heparin remains more important than N-sulfation using the HepaRG-based *in vitro* infection (Fig. S4).

In summary, heparin at the physiological concentration in the blood stimulated HBV infection *in vitro*.

## Discussion

The HepG2-NTCP system provides a new platform for the studies on the life cycle of HBV^7–9^. We established a similar HepG2-NTCP-AS system to search for potential host factors in the plasma involved in viral entry. To validate the *in vitro* infection system, we need to exclude the possibility of input virus contamination. In a time course experiment, we observed a reproducible trend of increasing signals of secreted HBsAg, HBeAg, and HBV DNA replicative intermediates. In addition, treatment with nucleoside analogs abolished these signals, suggesting that *de novo* synthesis post-viral entry is responsible for the generation of HBV specific proteins and DNA genome.

Heparan sulfate is known to serve as an attachment receptor for many bacteria, viruses, and parasites^27^. Many human viruses can initiate their infection by binding to cell surface heparan sulfate^28^. Heparin is structurally related to heparan sulfate, and can be produced by basophils and mast cells^13^. The best known function of heparin is to prevent blood from clotting. In most *in vitro* viral infection systems, soluble heparin was used as an inhibitor of infection at concentrations ranging from 1 μg/ml to 100 μg/ml, depending on the specific virus in each study^28^. On the other hand, heparin can also promote AAV-induced T cell activation and increase the uptake of AAV2 by dendritic cell^29^. In addition, heparin binding can induce conformational change at AAV2^30^.

Our studies on human plasma led to the identification of heparin as an enhancement host factor for *in vitro* infection with HBV. In earlier reports, heparin has been commonly used as an control inhibitor for HBV infection (Table 1). The opposite effects of heparin on HBV infection between our current study and earlier reports can be related to a number of possibilities: 1) We detected heparin enhancement at physiological concentration (1–5 µg/ml), while earlier reports observed heparin inhibition at much higher concentrations (25 U/ml, 100 µg/ml, or 100 IU/ml)^12,21,22^. In our heparin dose-response experiment, we observed no enhancement at 13.5 µg/ml, and strong inhibition at 40 and 120 µg/ml (Fig. 2 d).2) Earlier reports used 4% PEG in their infection procedure^12,21,22^. It was shown that PEG can enhance the binding between the glycosaminoglycan (GAG) side chain of HSPG and the PreS1 domain of HBV large envelope protein (L-HBsAg)^12^. In our studies, we observed heparin enhancement only at no PEG or 1.2% PEG. At 4% PEG, we observed no heparin enhancement either (Fig. 3d). 3) One earlier report pre-treated primary Tupaia hepatocytes with heparin at 5 µg/ml for 1 hour before HBV infection, and observed 50% inhibition on HBV infection efficiency^20^. The difference in the heparin effects here could include differences between primary Tupaia hepatocytes and human HepG2-NTCP-AS cells, the sources of input viruses (purified vs. non-purified), as well as different protocols for the heparin treatment (1 hr vs. overnight), HBV binding and incubation time. 4) Another earlier report used 9.4 µg/ml heparin in their infection protocol, and obtained 50% inhibition of infection efficiency^12^. We noted that this report used 4% PEG and the source of HBV was purified from the medium of an HBV-producing cell line HepG2.2.15. In our low-PEG or PEG-free assay system, the source of HBV was directly from the unpurified human serum. Overall, the effect on the infection efficiency from our low-heparin and low/no-PEG protocol, is less strong than the effect from the 4% PEG protocol. However, our system is more closely related to human natural infection. It is our hope that such a system could provide a platform for the search of host factors important in viral entry.

**Table 1:** Effects of heparin concentration on HBV *in vitro* infection.

Literature	MOI	Heparin dose	Virus source	PEG	Incubation time	Cell system	Effect
Iwamato *et al*., 2014^22^	1.8*10^4^	100IU/ml^*^	HepAD38	4%	16 h	HepG2-NTCP-C4	50% Inhibition
Watashi *et al*., 2014^21^	6000	25U/ml^*^	HepAD38	4%	16 h	HepaRG	100% Inhibition
Leistner *et al*., 2008^20^	100	100 µg/ml	Purified HBV from carriers	N/A	Pretreatment of heparin 1 h + 4 h HBV binding	PTH	100% Inhibition
Schulze *et al*., 2007^12^	100x concentrate of supernatant	100 µg/ml	HepG2.2.15	4%	16 h	HepaRG	100% Inhibition
**This study**	300–3000	1–5 µg/ml	HBe + and HBe- human sera	0–1.2%	24 h	HepG2-NTCP-AS	**2–3 fold stimulation**

^*^1 U/ml is similar to 1 IU/ml^35^. 1 U/ml = 6 µg/ml (Sigma Co.).

**Figure 5:**
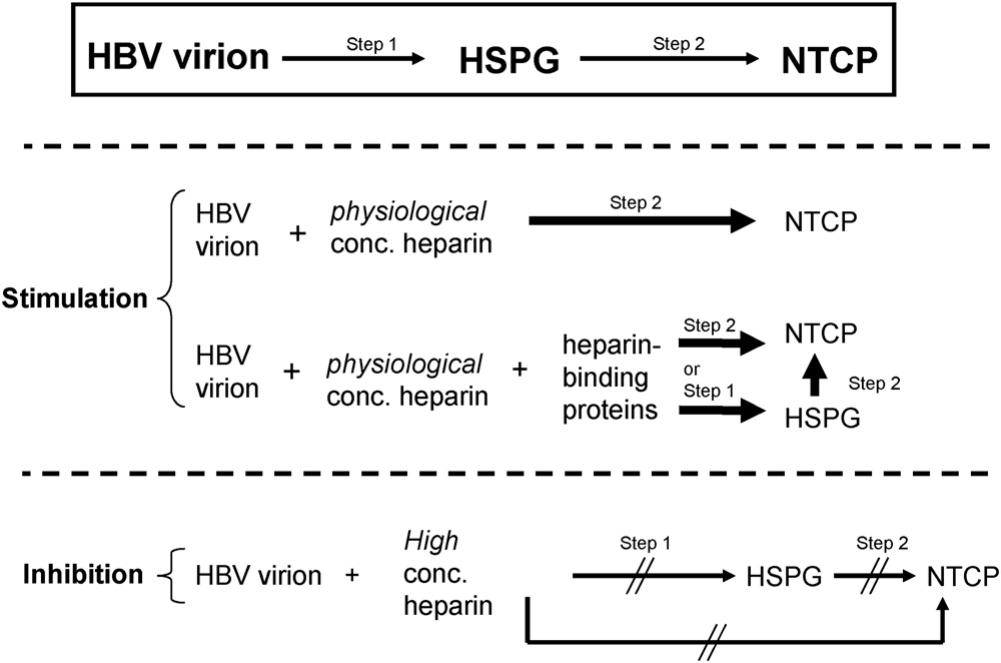
Hypothetical mechanisms for the effects of heparin concentration on HBV entry via a two-step process. Dotted lines separate Fig. 5 into three panels. *Upper panel*: It is generally believed that HBV entry would undergo a 2-step process (see references in Table 1). In step 1, HBV virions would bind to heparin sulfate proteoglycan (HSPG) on the surface of hepatocytes^12,20,25^. This initial attachment step is then followed by step 2, where the PreS1 peptide of the L-HBsAg would undergo some kind of conformational change (more exposed), and become more “activated” for binding to a bile salt transporter NTCP on the cell surface, leading to the internalization of HBV^25^. *Middle panel*: Heparin and heparan sulfate share similar structures^18^. To explain the stimulatory effect of heparin at physiological concentration (1–5 μg /ml), we postulate here that once soluble heparin binds to the virion surface, it can also activate the PreS1 domain of L-HBsAg. In this scenario, step 1 is no longer necessary for HBV binding to NTCP, and can be bypassed. There are many heparin-binding protein factors in the serum^19^. It is possible that some unknown heparin binding factor(s) could play a positive role in this viral entry cascade. For example, complex formation of virions, heparin, and heparin-binding proteins, could facilitate either the conventional step 1-binding to HSPG, or the direct binding to NTCP. *Lower panel*: At non-physiologically high concentrations of heparin, the surface of HBV virions are fully decorated with excessive amounts of heparin. In this case, virions saturated with negatively-charged heparin cannot bind to negatively charged HSPG. We also speculate here that overly heparinated HBV virions cannot activate the PreS1 domain ligand for the NTCP receptor recognition.

What could then be the mechanisms for heparin enhancement and inhibition on the *in vitro* HBV infection? It is generally believed that HBV can bind to HSPGs as a low-affinity receptor before the initiation of HBV infection^12,20,25,31^ (Fig. 5, upper panel). Heparin can bind to the large envelope protein of HBV (L-HBsAg)^12^. Presumably, higher concentration of heparin can bind to the L-HBsAg on the virion surface and interfere with HBV attachment to HSPGs on the surface of hepatocytes. Recently, it was reported that, in addition to the PreS1 domain, the heparin binding site of HBV envelope is another determinant of HBV infectivity^25^. The heparin binding site is located close to an antigenic loop (AGL), which is separated from the PreS1 domain, and is structurally related to the group *a*-determinant of HBsAg. It is tempting to speculate that lower concentration of heparin can bind to the HBV virion surface and induce an active conformation, leading to the bypass of HSPG attachment, and thus allow more direct and effective interactions between the heparin-virion complex and the NTCP receptor (Fig. 5, middle panel). According to this conceptual framework, excessive amount of heparin binding could dampen the activation of the PreS1 domain, and prevent it from interaction with NTCP (Fig. 5, lower panel). Further studies would be required to elucidate the mechanism of heparin enhancement in molecular details.

In this regard, it is worth mentioning that we observed no significant enhancement on HBV infection by using non-physiological compounds, such as chondroitin sulfate and dextran sulfate (Fig. S5). In addition, in our preliminary studies, we found that heparin can significantly enhance HBV binding to HepG2-NTCP-AS hepatocytes in a 4-hr incubation period at 4 °C or 37 °C by 4% PEG, but not by low (1.2%) or no PEG (Fig. S6a). On the contrary, in a 24-hr incubation period at 37 °C condition, significant heparin-mediated enhancement on HBV binding to hepatocytes, can be more easily detected at low or no PEG than 4% PEG (Fig. S6b).

We noted that the O-sulfation of heparin is more important for the infection enhancement than N-sulfation (Figs S3 and S4). It is possible that O-sulfated heparin has a higher affinity for HBV virion than N-sulfated heparin. Alternatively, it is equally possible that O-sulfated heparin can recruit more or different host factors for infection. When completely de-N-sulfated heparin was studied for its inhibitory effect on HBV infection, no inhibition was observed^12^. Therefore, by inference, it is likely that O-sulfated heparin does not inhibit HBV infection, even when given in excess. Taken together, O-sulfated heparin may be considered as a positive factor for HBV infection.

In conclusion, we found that heparin at physiological concentration has a moderate, yet highly reproducible, stimulatory effect on HBV infection in the low or no PEG condition. Relative to the enhancement effect of 4% PEG, heparin enhancement is less potent. However, one merit from our study here resides in the establishment of a PEG-free HepG2-NTCP-AS platform, which is more closely mimicking HBV natural infection *in vivo*. It will be worth investigation in the future whether any of the hundreds of heparin-binding host factors could play a role in viral entry in the liver.

## Methods

### Ethics statement

Informed consent was obtained from patients. The study protocols were approved by the Institutional Review Board of the College of Public Health, National Taiwan University, Taipei, Taiwan and Kaohsiung Medical University Hospital, Kaohsiung, Taiwan (IRB number is KMUH-IRB950134). All methods in this study were performed in accordance with the relevant guidelines and regulations.

### Reagents

CMV6-NTCP-flag tag expression vector was purchased from Origene.

Heparin, DeO heparin, DeN heparin and anti-flag M2 antibody were purchased from Sigma-Aldrich. PNGase F was purchased from Promega. PreS1 peptide was synthesized by Yao-Hong Biotechnology Inc. (Taiwan). PEG6000 was purchased from Millipore corporation. 30% stock solution was prepared in PBS buffer.

### Establishment of HepG2-NTCP-AS cell line

Parental HepG2 cells were transfected with CMV6-NTCP-flag tag expression plasmid. Transfected cells were selected at 400 μg/ml Neomycin. Neo-resistant colonies were amplified and examined for NTCP expression by Western blot using anti-Flag M2 antibody. Colonies expressing a high level of NTCP were tested for HBV infection. HBV infection was evaluated by HBsAg, HBeAg ELISA, IFA of HBc, and Southern blot analysis.

### Plasma fractionation

Normal human plasma was fractionated by Amicon Ultra Centrifugal Filters. A total of 2 ml normal human plasma was fractionated serially through 100 kD, 10 kD, and 3 kD filters. Concentrated fractions on each filter were diluted up to 10 ml with PBS, and the centrifugation process was repeated to further enrich plasma proteins in each MW fraction. Each concentrated fraction was adjusted up to 2 ml final volume by PBS, and stored at −80 °C. For HBV infection, plasma fractions were added into the infectious serum sample at a 10% final concentration (v/v).

### HBV *in vitro* infection assay

HepaRG cells were purchased from Invitrogen and cultured according to the product manual. HepG2-NTCP-AS cells were maintained in DMEM supplemented with 10% FBS, 100 U/ml penicillin and 100 μg/ml streptomycin. Before infection, HepG2-NTCP-AS cells were maintained in serum-free DMEM supplemented with 5 μg/ml transferrin, 3 μg/ml insulin, 5 ng/ml sodium selenite, 2 mM L-glutamine, 100 U/ml penicillin, 100 μg/ml streptomycin and 2% DMSO for 1–2 days. Approximately 10^5^ HepG2-NTCP-AS cells in one well of a 24-well plate were infected by inoculating a total of 0.5 ml/well of serum-free DMEM medium supplemented with HBV serum (300–3000 MOI), 1.2–4.0% PEG and 2% DMSO. PreS1 peptide treatment was conducted by incubating 500 nM PreS1 peptide 30 minutes before and during HBV infection. After HBV infection at 37 °C for 24 hrs, cells were washed with PBS twice at day 1 and day 3 post-infection. Cells were maintained in DMEM medium supplemented with 5% FBS and 2% DMSO after infection. Culture media were collected every other day starting from day 5 post-infection.

### Western blot

NTCP-flag protein expression was detected by anti-flag M2 antibody from Sigma-Aldrich. Western blot assay procedures were performed as described previously^32^.

### Immunofluorescence assay

HBc protein was stained by rabbit anti-HBc as reported previously^32^. Immunofluorescence assay procedures were performed as detailed elsewhere^33^.

### Southern blot assay

HBV whole genomic DNA was purified from HBV-monomer plasmid DNA by EcoRI digestion. HBV DNA probe was radiolabeled by Red Prime DNA labeling system according to the manufacturer’s protocol (GE Healthcare). The Southern blot assay was performed according to the standard protocol as described previously^34^. The typical smearing signals on the Southern blot reflect characteristic HBV DNA replicative intermediates with different molecular weights, and are not due to overexposure on the Typhoon Imager or X-ray film.

### HBsAg and HBeAg detection by ELISA

HBsAg and HBeAg ELISA kits were purchased from General Biological Corporation (Taipei, Taiwan). Determinations of HBsAg and HBeAg in the medium were performed according to the manufacturer’s protocol.

### HBV binding assay

HepaRG cells were maintained following the product manual. HepG2-NTCP-AS cells were maintained in DMEM medium supplemented with 2% DMSO for 1 day before HBV inoculation. Human HBV serum (4%) was incubated with HepG2-NTCP-AS and HepaRG cells at 4 °C or 37 °C for 4 and 24 hours. After incubation, cells were washed extensively with ice cold PBS 5 times. Cell-bound HBV DNA was extracted together with cellular genomic DNA by Roche High Pure Viral Nucleic Acid Kit according to the manufacturer’s protocol. HBV binding to hepatocytes was counted by HBV copy number per well, using quantitative Real time PCR assay and HBV core specific primers “HBV-2279-F: TTCGCACTCCTCCAGCTTAT, HBV-2392-R: GAGGCGAGGGAGTTCTTCTT”. HBV copy number was calculated by comparing to the known amount of HBV monomer plasmid DNA. Quantitative real time PCR assay was performed by the 7500 real time PCR system using POWER SyBr green dye (Applied Biosystems).

## Electronic supplementary material

Supplementary Information
