# Supplementary material for: Heparin at physiological concentration can enhance PEG-free in vitro infection with human hepatitis B virus
Source: Sci Rep. 2017 Oct 31;7:14461. doi: 10.1038/s41598-017-14573-9 (PMC5663848; doi:10.1038/s41598-017-14573-9)
Supplement: Supplementary file 1 — Supplementary Information [file 41598_2017_14573_MOESM1_ESM.pdf]

# **Heparin at physiological concentration can enhance PEG-free *in vitro* infection with human hepatitis B virus**

Authors: Gansukh Choijilsuren[1,2,3#], Ren-Shiang Jhou[2#], Shu-Fan Chou[2], Ching-Jen Chang[2], Hwai-I Yang[4], Yang-Yuan Chen[5], Wan-Long Chuang[6], Ming-Lung Yu[6], and Chiaho Shih[2*]

[1]Taiwan International Graduate Program in Molecular Medicine, National Yang-Ming University and Academia Sinica, Taipei, Taiwan. [2]Institute of Biomedical Sciences, Academia Sinica, Taipei, Taiwan. [3]Institute of Biochemistry and Molecular Biology, National Yang-Ming University, Taipei, Taiwan. [4]Genomics Research Center, Academia Sinica, Taipei, Taiwan. [5]Changhua Christian Hospital, Changhua, Taiwan. [6]Hepatobiliary Division, Department of Internal Medicine, Kaohsiung Medical University Hospital, Kaohsiung, Taiwan.

# equal contribution

* To whom correspondence should be addressed

Mailing address: Institute of Biomedical Sciences, Academia Sinica, Taipei, Taiwan

Tel: 886-2-2652-3996; Fax: 8862-2652-3597; E-mail: cshih@ibms.sinica.edu

## Supplementary Information

### Fig. S1

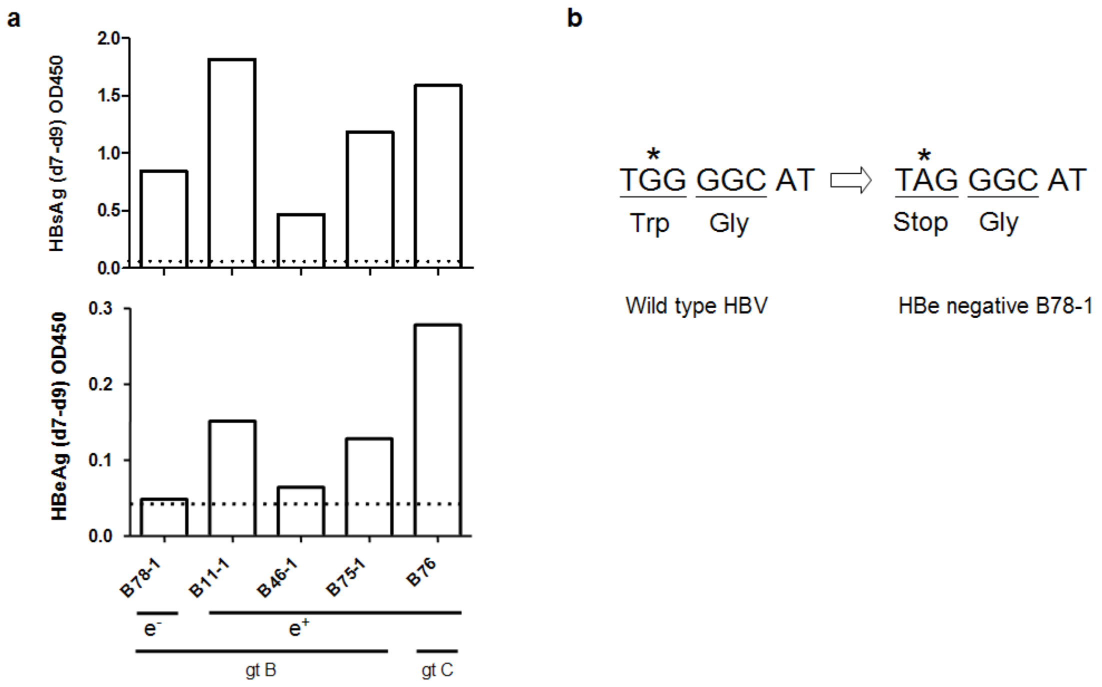


**HBV from both HBeAg positive and negative patients' serum is infectious *in vitro*.**

(a) Serum samples from different human hepatitis B patients (Supplementary Table S1) successfully infected HepG2-NTCP-AS cells in the presence of 4% PEG. Both HBsAg and HBeAg were measured by ELISA at 7-9 days post-infection (dpi). B78-1 is from an HBeAg negative carrier. B76 contains HBV genotype C (gt C).

(b) A stop codon mutation was found in the pre-C region of HBV sequences in HBeAg-negative patient B78-1. Asterisk (*) indicates the site of a G-to-A mutation at nt 1896 (*ayw* numbering system). Dotted lines represent the cutoff values of HBsAg and HBeAg ELISA assay.

**Fig. S2**

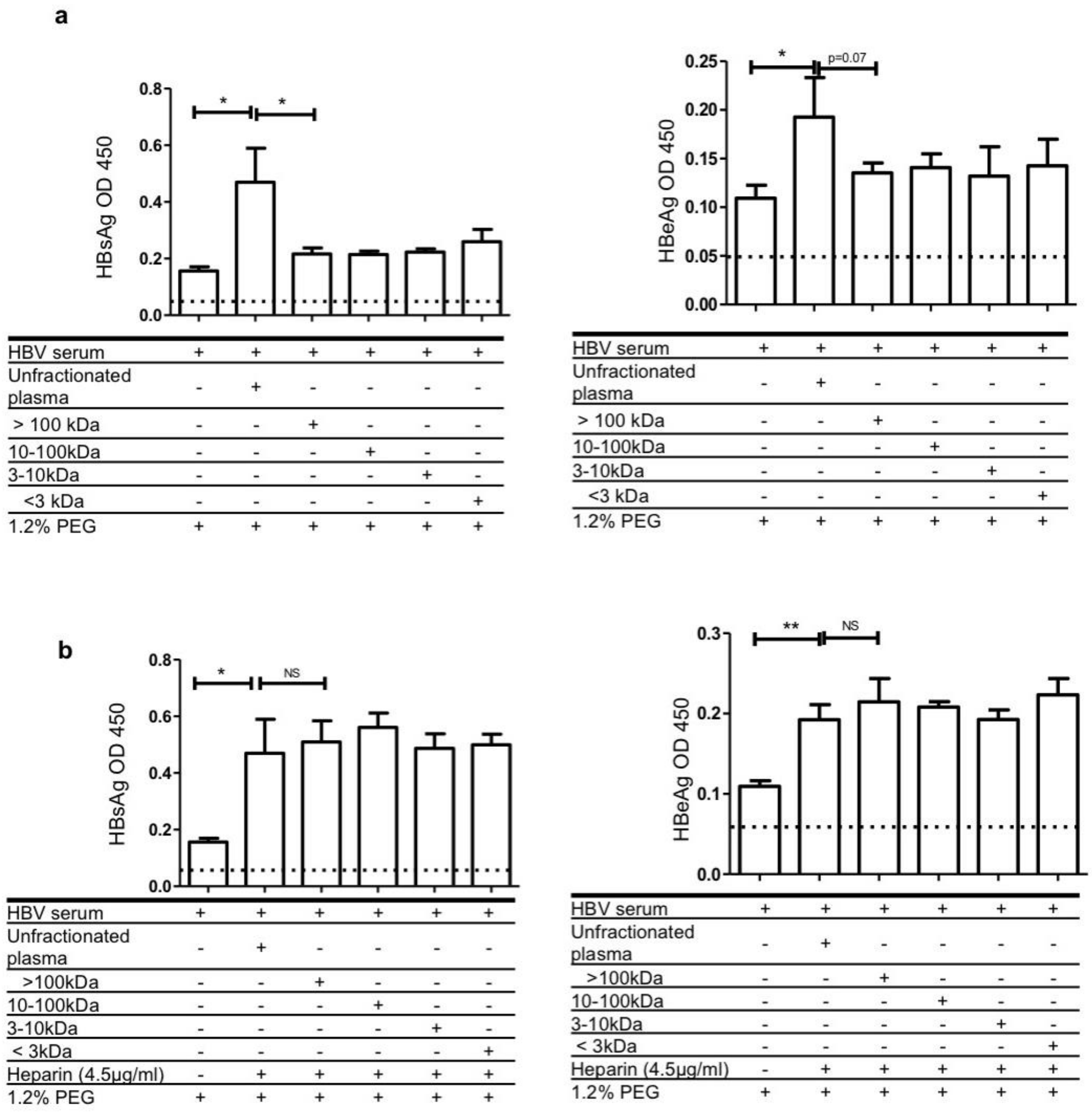


**Heparin is the principal ingredient in human plasma to the enhancement of HBV infection.**

(a) Different-sized fractions of human plasma (Materials and Methods) had no effect on HBV infection. ELISA of HBsAg (left panel) and HBeAg (right panel)

(b) Significant enhancement of HBV infection was observed by heparin supplement to every individual fractions of human plasma.

**Fig. S3**

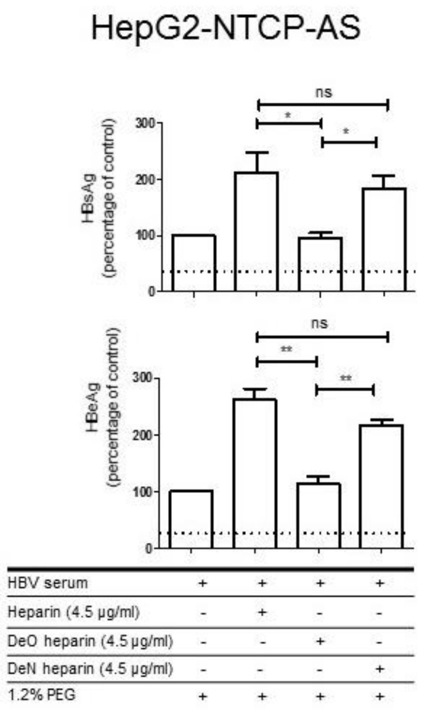


**O-sulfation of heparin is required for its enhancement on HBV infection in HepG2-NTCP-AS cell system.**

Comparison of the enhancement effects on HBV infection between 4.5 μg/ml DeO-sulfated heparin and DeN-sulfated heparin by ELISA. The relative levels of HBsAg and HBeAg by ELISA are averages from three different experiments. The levels of HBsAg and HBeAg from heparin-free infection experiment are used here as a reference (defined as 100%). $*p < 0.05$, $**p<0.01$

**Fig. S4**

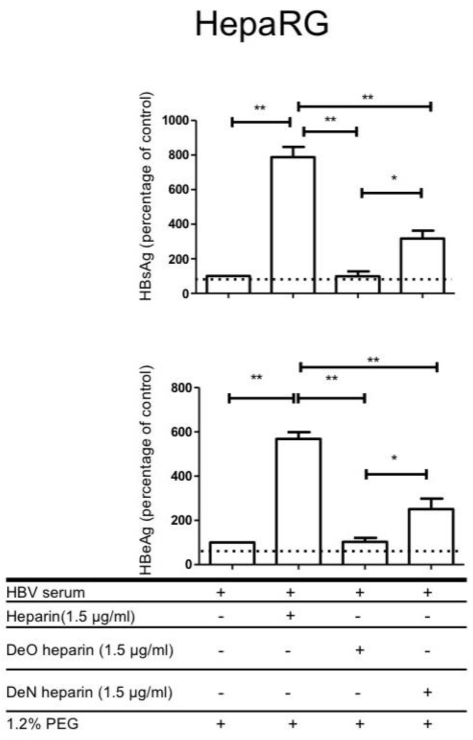


**O-sulfation is also important for heparin enhancement of HBV infection in the HepaRG cell system**. *$p < 0.05$, **$p<0.01$

**Fig. S5**

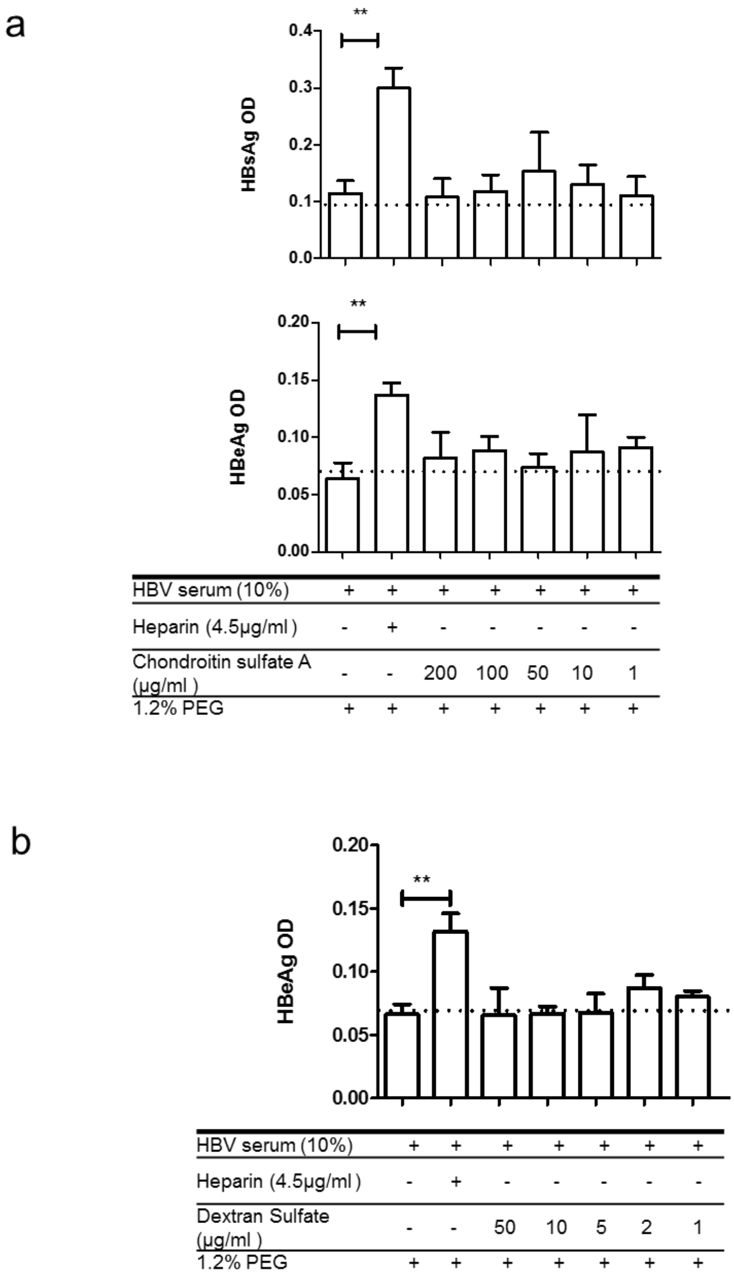


**No significant effect on HBV infection by treatments with dextran sulfate or chondroitin sulfate A**.

**Fig. S6**

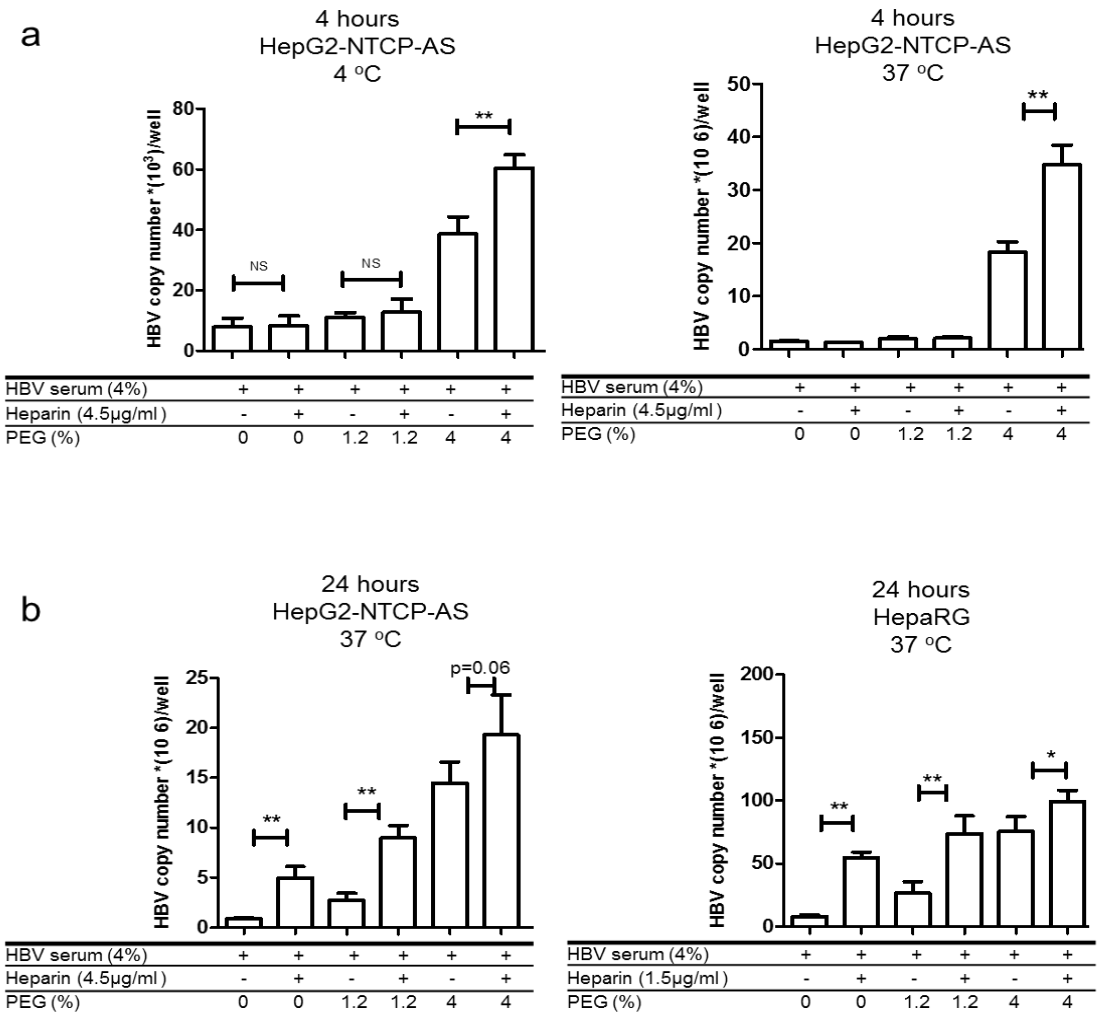


**Potential effect of heparin on HBV binding were assayed by incubation of human serum-derived HBV particles and the adhering HepG2-NTCP-AS cells or HepaRG cells, at different temperatures (4$^0$C or 37$^o$C) for different periods (4 hr or 24 hr).**

(a) No significant heparin effect on HBV binding to HepG2-NTCP-AS cells at no or low PEG concentration in 4-hr incubation. In contrast, 4% PEG had a strong effect on binding. (b) Heparin can enhance binding to HepG2-NTCP-AS or HepaRG cells at 37$^o$C for 24 hr at no or low PEG.

**Table S1.** Clinical data of serum samples from Taiwanese HBV patients

| Patient ID | Gender | Age | Viral DNA* GE/ mL | Viral genotype | HBsAg@ | HBeAg@ |
|---|---|---|---|---|---|---|
| B78-1 | M | 40 | $2.31x10^{9}$ | B | + | - |
| B11-1 | M | 23 | $1.43x10^{9}$ | B | + | + |
| B46-1 | F | 37 | $1.28x10^{9}$ | B | + | + |
| B75-1 | M | 52 | $8.1x10^{8}$ | B | + | + |
| B76# | M | 34 | $5x10^{9}$ | C | + | + |

*Determined by RT qPCR
@ ELISA cut-off value=0.1
# Sample B76 was used in most of the current studies. The HBV titer fluctuated overtime ($10^{8}$-$10^{9}$) in patient B76. Therefore, the viral DNA titer here reflects the amount of virus harvested at a particular time point.
